# A phase I–II controlled randomized trial using a promising novel cell-free formulation for articular cartilage regeneration as treatment of severe osteoarthritis of the knee

**DOI:** 10.1186/s40001-018-0349-2

**Published:** 2018-10-24

**Authors:** Ivan Delgado-Enciso, Juan Paz-Garcia, Jose Valtierra-Alvarez, Jorge Preciado-Ramirez, Roman Almeida-Trinidad, Jose Guzman-Esquivel, Martha A. Mendoza-Hernandez, Alberto Garcia-Vega, Alejandro D. Soriano-Hernandez, Jose L. Cortes-Bazan, Hector R. Galvan-Salazar, Ariana Cabrera-Licona, Iram P. Rodriguez-Sanchez, Margarita L. Martinez-Fierro, Josuel Delgado-Enciso, Brenda Paz-Michel

**Affiliations:** 1 Instituto Estatal de Cancerologia, Colima State Health Services, 28000 Colima, Mexico; 2 School of Medicine, University of Colima, 28030 Colima, Mexico; 3 Centro Hospitalario Union, Villa de Álvarez, 28970 Colima, Mexico; 4 Hospital Regional Universitario, Colima State Health Services, 28019 Colima, Mexico; 5 Hospital General de Zona No. 1 IMSS, Villa de Álvarez, 28983 Colima, Mexico; 6 Esteripharma México, S.A. de C.V, Patricio Sanz 1582, Colonia del Valle Centro, 03100 Ciudad de México, Mexico; 7 School of Biological Sciences, Universidad Autonoma de Nuevo Leon, 64460 Monterrey, Nuevo León Mexico; 8 Molecular Medicine Laboratory, Academic Unit of Human Medicine and Health Sciences, Universidad Autónoma de Zacatecas, 98160 Zacatecas, Mexico; 9 Foundation for Cancer Ethics, Education and Research of the Cancerology State Institute, 28085 Colima, Mexico

**Keywords:** Osteoarthritis, Knee, Treatment, Cartilage regeneration, Chondrocytes, Stem cell, Clinical trial, Arthroplasty, Nonsteroidal anti-inflammatory drugs, Mesenchymal cells, Chondrogenesis

## Abstract

**Background:**

A promising novel cell-free bioactive formulation for articular cartilage regeneration, called BIOF2, has recently been tested in pre-clinical trials. The aim of the present study was to evaluate the efficacy and safety of BIOF2 for intra-articular application in patients with severe osteoarthritis of the knee.

**Methods:**

A prospective, randomized, 3-arm, parallel group clinical trial was conducted. It included 24 patients with severe osteoarthritis of the knee (WOMAC score 65.9 ± 17). Before they entered the study, all the patients were under osteoarthritis control through the standard treatment with nonsteroidal anti-inflammatory drugs (NSAIDs), prescribed by their family physician. Patients were distributed into three groups of 8 patients each (intra-articular BIOF2, total joint arthroplasty, or conservative treatment with NSAIDs alone). The WOMAC score, RAPID3 score, and Rasmussen clinical score were evaluated before treatment and at months 3, 6, and 12. BIOF2 was applied at months 0, 3, and 6. Complete blood count and blood chemistry parameters were determined in the BIOF2 group before treatment, at 72 h, and at months 1, 3, 6, and 12. In addition, articular cartilage volume was evaluated (according to MRI) at the beginning of the study and at month 12.

**Results:**

The NSAID group showed no improvement at follow-up. Arthroplasty and BIOF2 treatments showed significant improvement in all the scoring scales starting at month 3. There were no statistically significant differences between the BIOF2 group and the arthroplasty group at month 6 (WOMAC score: 19.3 ± 18 vs 4.3 ± 5; *P* = 0.24) or month 12 (WOMAC score: 15.6 ± 15 vs 15.7 ± 17; *P* = 1.0). Arthroplasty and BIOF2 were successful at month 12 (according to a WOMAC score: ≤ 16) in 75% of the patients and the daily use of NSAIDs was reduced, compared with the group treated exclusively with NSAIDs (RR = 0.33, 95% CI 0.12–0.87, *P* = 0.02. This result was the same for BIOF2 vs NSAIDs and arthroplasty vs NSAIDs). BIOF2 significantly increased the articular cartilage by 22% (26.1 ± 10 vs 31.9 ± 10 cm^2^, *P* < 0.001) and produced a significant reduction in serum lipids. BIOF2 was well tolerated, causing slight-to-moderate pain only upon application.

**Conclusions:**

The intra-articular application of the new bioactive cell-free formulation (BIOF2) was well tolerated and showed no significative differences with arthroplasty for the treatment of severe osteoarthritis of the knee. BIOF2 can regenerate articular cartilage and is an easily implemented alternative therapy for the treatment of osteoarthritis.

*Trial registration* Cuban Public Registry of Clinical Trials (RPCEC) Database RPCEC00000250. Registered 08/15/2017—Retrospectively registered, http://rpcec.sld.cu/en/trials/RPCEC00000250-En.

**Electronic supplementary material:**

The online version of this article (10.1186/s40001-018-0349-2) contains supplementary material, which is available to authorized users.

## Background

Osteoarthritis is a chronic disorder of the synovial joints. There is progressive softening and disintegration of articular cartilage [1], and it is the most common form of arthritis. The prevalence of osteoarthritis is on the rise and will increase in the coming decades, due to longevity and the growing prevalence of obesity [2]. By the age of 65, approximately 80% of the population may present with the disease [3]. The knee joint is the most frequently affected, followed by the hip and hand joints [4]. It is the primary cause of disability and impaired quality of life in the elderly [3]. In addition to producing intense pain, it affects mobility, mood, and sleep patterns, and reduces physical fitness, which can result in an increased risk of cardiometabolic comorbidity [3, 5–7].

Despite the considerable medical necessity, no treatment has yet been proven to act as a disease-modifying agent that can halt or reverse the structural progression of osteoarthritis. Apart from education and exercise, the only available non-surgical treatments are directed at symptoms—primarily alleviating pain and enhancing daily activities and quality of life [8, 9].

When conservative therapy is ineffective in severe cases of osteoarthritis of the knee, total joint arthroplasty, in which the joint is substituted with a prosthesis, is recommended. However, it is an expensive surgical treatment [10] and often entails unacceptably long waiting periods. The risk for serious adverse events following total joint arthroplasty is low, but perioperative risks can be high in elderly patients or those with comorbidities. Good outcome in total joint arthroplasty is achieved in only half of patients presenting with multiple troublesome joints and comorbidity, and arthroplasty is not recommended in young patients, because the artificial implant has a finite lifespan (usually 10–15 years) [11]. Therefore, it is a procedure that should only be carried out after a rigorous risk–benefit assessment [12].

In recent years, there has been a search for new pharmacologic therapy regimens. Antidepressants, nonsteroidal anti-inflammatory drugs (NSAIDs), and opiates are administered orally, as well as other substances, whose benefits have been limited or null, such as glucosamine, chondroitin sulfate, methylsulfonylmethane, or collagen hydrolysates. Intra-articular injections of corticosteroids produce only short-term benefits of significant improvement. Hyaluronic acid derivatives have apparent effectiveness between 5 and 13 weeks after treatment. They offer lower efficacy than steroids in the short term, but their benefits may increase over a period of weeks and can be effective for up to 6 months [13]. Platelet-rich plasma (PRP) is being considered as an innovative and promising tool, with an effectiveness pattern comparable to that of the intra-articular administration of hyaluronic acid [14]. The use of scaffolds alone, or in combination with stem cells or gene therapy, is strategies that are currently under development [1, 11].

An innovative concept is the creation of novel cartilage in a damaged joint through the administration of cell-free bioactive substances that promote chondrogenesis. That idea is based on the fact that the fluid inside the joint contains mesenchymal cells (MSCs) that are able to differentiate into chondrocytes [15].

A promising novel bioactive formulation for articular cartilage regeneration, called BIOF2, has recently been tested in pre-clinical trials [16]. The intra-articular application of BIOF2 significantly increased cartilage thickness (12–38%) in various animal models, compared with articular cartilage treated with saline [17]. In addition, the articular area and number of chondrocytes increased significantly, maintaining an unaltered chondrocyte/mm^2^ proportion. Evaluation of the histologic architecture also displayed a decrease in the grade of articular damage in the animals treated with BIOF2 [17]. Therefore, the proposed hypothesis is that the intra-articular application of BIOF2 in humans can induce cartilage formation in joints with severe osteoarthritis, resulting in a safe alternative to total joint arthroplasty. The aim of the present study was to evaluate the efficacy and safety of the intra-articular application of BIOF2, in patients with severe osteoarthritis of the knee, through a randomized, prospective, and comparative study.

## Methods

### Study design

We conducted a prospective, randomized, simple-blind, 3-arm, parallel group, phase I–II clinical trial between November 2015 and October 2017. The study was carried out according to the “CONSORT statement” guidelines for randomized controlled trials.

The present study was approved by the ethics committee of the Cancerology State Institute of the Health Services of the State of Colima, Mexico, and written informed consent was obtained from all the participants. The present clinical trial was registered as ARTROTX: RPCEC00000250 in the Cuban Public Registry of Clinical Trials (RPCEC) Database. The RPCEC trial registration data set is part of the data set of the International Platform Registry as established by the World Health Organization (WHO) and the International Committee of Medical Journal Editors.

### Study subjects

The following inclusion criteria were used: patients ≥ 50 years of age, with a body mass index (BMI) ≤ 35 kg/m^2^, and presenting with osteoarthritis in one knee, according to the diagnostic criteria of the American College of Rheumatology [18], with a Western Ontario and McMaster Universities Osteoarthritis Index (WOMAC) score > 39, despite conservative therapy [19]. A WOMAC score ≥ 39 has been reported as an appropriate criterion for total knee replacement [20]. To be included in the trial, patients had to be under the standard conservative treatment (NSAIDs) by their family physician, with significant symptoms and/or functional limitations associated with reduced health-related quality of life. The following exclusion criteria were used: having undergone intra-articular treatment within 12 months prior to the study, a history of knee trauma or knee surgery, *genu varum* or *genu valgum* malalignment (greater than 20°), inflammatory polyarthritis or fibromyalgia or chronic fatigue syndrome, thromboembolic disease, or hemorrhagic blood disease; Hb < 80 g/L; neuromuscular disease, cancer, or metabolic bone disease; alcoholism and/or drug addiction; or an American Society of Anesthesiologists anesthesia rating > 3. The participants were recruited from a secondary healthcare center (*Centro Hospitalario Union* in the city of Villa de Álvarez) located in the State of Colima, Mexico.

Twenty-four patients were allocated to the intra-articular BIOF2 group, the total joint arthroplasty group, or the group continuing with the standard conservative treatment (NSAIDs) prescribed by their family physician. Randomization was performed using a computer-generated set of scratch cards, with blocks of 8 and a 1∶1:1 ratio for each arm, and patients were assigned to one of the 3 groups. The researchers conducting that process did not participate in the evaluation of the results. It should be emphasized that all the patients were under osteoarthritis control through the standard treatment with NSAIDs prescribed by their family physician before they entered the study.

### Arthroplasty procedure

All surgeries were performed by the same skilled surgical team and under subarachnoid anesthesia. A medial parapatellar approach was used to expose the knee joint. An inflatable tourniquet was attached to the limb with a pressure of 100 mmHg above the systolic blood pressure. An intramedullary alignment jig was used for the distal femoral resection, and an extramedullary device was used for the tibia. The implant employed was a cemented total knee prosthetic component (Vanguard Knee System Biomet, Warsaw, Indiana, USA) with no patellar resurfacing that substituted the posterior cruciate ligament. In the treatment group, the femoral hole was sealed with an autologous bone plug obtained from bone off-cuts and cement. Subcutaneous skin closure was then performed. The tourniquet was deflated, after application of a compressive elastic bandage. A dose of 40 mg of enoxaparin was administered subcutaneously to all patients at 22 h post-surgery and then daily, until discharge. The patients had a drainage tube that was removed 24 h after the procedure. Active isometric quadriceps movements, straight leg raises, and extension–flexion motions were encouraged 48 h after the surgery. All patients were released from the hospital at 48–72 h after surgery. The patients were programmed for transfusion if their Hb levels dropped below 80 g/L with symptoms of syncope, fatigue, and/or palpitations. The skin sutures were removed 10 days after surgery and walking with the aid of a walker was indicated. The patients were referred to the physiotherapy and rehabilitation service. They continued to see their family physicians for general care, healthy lifestyle promotion, and when necessary, to continue with NSAID pharmacologic treatment. The researchers did not intervene in the prescription of drugs or lifestyle indications.

### BIOF2 administration

BIOF2 is a patented formulation composed of a corticosteroid, a type of insulin, and organic acids, whose intra-articular application is intended to stimulate cartilage regeneration. The BIOF2 manufacturing process was performed according to Good Manufacturing Practices (GMP) for pharmaceutical products for use in clinical trials by Esteripharma Mexico (Mexico City, Mexico).

BIOF2 was administered on three occasions, with 3-month intervals (at months 0, 3, and 6). It was an outpatient application performed at the traumatology and orthopedics consultation office. BIOF2 treatment was administered as an injection into the knee joint space under sterile prep conditions, (i.e., prior to injection, the knee was cleaned with an antiseptic). The patient was in a seated position, with the knee undergoing treatment flexed at 0°. The area of injection was inferior lateral to the patella at the lateral level of the joint line. The principal investigator decided whether it was appropriate to apply local anesthesia with lidocaine. A 20-gauge needle 1.5 in long was used for the injection. The needle was passed through the fat pad to the firm surface of the intercondylar notch. Following needle withdrawal, pressure was applied with a cotton ball with alcohol at the injection site, which was then covered with a sterile dressing (BandAid). The patients continued to carry out their daily activities after the procedure, with no special indications, and were referred to the physiotherapy and rehabilitation service. They continued to see their family physicians for general care, healthy lifestyle promotion, and when necessary, to continue with NSAID pharmacologic treatment. The researchers did not intervene in the prescription of drugs or lifestyle indications.

#### Standard conservative treatment with NSAIDs

That patient group continued with their treatment as prescribed by their family physicians. Treatment consisted of NSAID administration and the promotion of a healthy lifestyle. The researchers did not intervene in the prescription of drugs or lifestyle indications. The patients were referred to the physiotherapy and rehabilitation service. It was written in the case record that the patients were candidates for arthroplasty and they were told they could opt for surgery at any time during the study follow-up, depending on their wishes and the possibility of access to government programs for knee replacement or by means of their own resources.

### Outcome measures and follow-up

The primary endpoint was the change in WOMAC score to ≤ 29 at months 3 and 6, and ≤ 16 points at month 12. Treatment of total joint arthroplasty, taken as the gold standard for treatment of severe osteoarthrosis of the knee, was considered successful with that score [21]. The WOMAC instrument has a total score and subscales for stiffness, pain, and physical function [22].

The secondary endpoints included:

- Change in the Routine Assessment of Patient Index Data 3 (RAPID3), which is a pooled index of the three patient-reported Core Data Set measures of the American College of Rheumatology: physical function, pain, and patient estimate of global status. The RAPID3 instrument has a final score of 0 to 10 that was interpreted as near remission (0–1), low severity (1.3–2), moderate severity (2.3–4), and high severity (4.3–10). Although it is mainly used in rheumatic diseases, it is considered to be useful for evaluating osteoarthritis [23, 24]. Treatment was considered successful with a score of 2 or less.
- Change in the Rasmussen clinical score. It provided a record of functional results of the joint after treatment. A score of 28–30 was excellent, a score of 24–27 was good, a score of 20–23 was fair, and a score of < 20 was poor [25]. Treatment was considered successful with a score of 24 or more and a change in the daily use of NSAIDs at 1 year of progression.
- Change in NSAID consumption. All the patients were under osteoarthritis control through the standard treatment with NSAIDs prescribed by their family physician before they entered the study. NSAID consumption during the entire study was monitored by the researchers through anamnesis.

The primary and secondary endpoints were determined at the baseline and at months 3, 6, and 12 after the beginning of treatment.

The area of articular cartilage of the knee was evaluated in the BIOF2 group before treatment and at month 12 of treatment, through three-dimensional cartilage reconstruction from magnetic resonance imaging (MRI) studies. MRI was performed using a clinical 1.5T magnet (Magnetom Expert; Siemens Healthcare GmbH, Erlangen, Germany) and a circular polarized transmit–receive extremity coil. To obtain high-contrast and high-resolution images of the cartilage, a T1-weighted spoiled 3D gradient-echo sequence was used (fast low-angle shoot sequence with selective water excitation, radiofrequency amplitude ratios 1-2-1, repetition time 19 ms, echo time 8.6 ms, flip angle 20°, and bandwidth 130 Hz/pixel). The partition thickness was 5 mm and the in-plane resolution was 0.31 mm (field of view 160 mm, matrix 5122 pixels, phase resolution 100%, and slice resolution 75%). One coronal, sagittal, and axial data set of the tibiofemoral joint and of the patellofemoral joint were acquired. The data were then transferred to a work station for analysis using the ECLIPSE Versinn 11.0 software, designed by Varian Medical Systems (Palo Alto, CA, USA). In all the axial MRI images, the bone and cartilaginous structures were first separately defined. Afterwards, the axial views were joined, interpolating one view with another, to create a continuous 3D structure of the region of the knee. The coronal and sagittal views of the joint were also reconstructed. The volume of the cartilage (cm^3^) was obtained using ECLIPSE algorithm tools.

In the BIOF2 group, the safety of the procedure was measured by the appearance of pain or serum marker alterations of: systemic inflammation (erythrocyte sedimentation rate, C-reactive protein, and fibrinogen), serum lipids (total cholesterol and triglycerides), complete blood count, and liver function tests or kidney function tests. Those parameters were evaluated at baseline, at 72 h, and at months 1, 3, 6, and 12. The delayed adverse effects of infection, muscular atrophy, profound venous thrombosis, hematoma, tissue hypertrophy, formation of adhesions, or systemic reactions, such as abdominal pain/discomfort, were evaluated in all groups.

### Blinding

Only the researchers that evaluated treatment effectiveness through the WOMAC, RAPID3, and Rasmussen clinical score instruments, those that carried out the anamnesis in relation with NSAID consumption, and the researchers that performed the statistical analyses were blinded.

### Sample size

The sample size calculation was based on the difference (60%) in the number of patients with a successful result (a WOMAC score ≤ 16) at 1 year, between the BIOF2 group and the group that received the standard conservative treatment with NSAIDs. Eight patients from each group were needed to reach the required power (0.8) when the statistical analysis was performed at the level of the two-tailed alpha (0.05). At the end of the study, the statistical power for detecting a difference between two different groups was calculated (alpha = 0.05), utilizing the number of patients with therapeutic success at 12 months between the BIOF2 group and the NSAID group, and its result was 96.4%.

### Statistical analysis

Data were presented as percentages or mean ± standard error or standard deviation. For the inferential statistics, normal data distribution was first determined using the Kolmogorov–Smirnov test and the equality of variances was confirmed using the Levene’s test. One-way ANOVA with Bonferroni’s post hoc test was employed to compare the numerical variables (with normal distribution) between the three groups (BIOF2, NSAIDs, and arthroplasty). The categorical values were compared using the Fisher’s exact test. The area of articular cartilage of the knee (MRI evaluations), serum markers, and complete blood counts of the patients in the BIOF2 group was compared before and after treatment, utilizing the paired Student’s *t* test. The treatment success percentage (WOMAC score ≤ 29 points at months 3 and 6 and ≤ 16 points at month 12; Rasmussen clinical score ≥ 24 points, and RAPID3 score ≤ 2 points) was calculated for the different treatments at months 3, 6, and 12. The comparison of two data series, such as cartilage volume before and after treatment, was carried out using the paired Student’s *t* test. The Pearson correlation coefficient (r) was employed to correlate articular cartilage volume or its increase in percentage with the WOMAC score, RAPID3 score, and the Rasmussen clinical score. The relative risk (RR) and 95% confidence interval were calculated to determine the probability of habitual NSAID use, comparing the NSAID group vs the BIOF2 group or arthroplasty group. The statistical analysis was performed using the SPSS software, version 20 (IBM Corp., Armonk, NY, USA), with the exception of the RR, which was calculated using the MedCalc v17.7.2 software (MedCalc Software bvba, Ostend, Belgium). A two-sided *P* < 0.05 was considered statistically significant.

## Results

Of the 58 patients that were screened, 24 were randomized and distributed into the three study groups (see Additional file 1). Table 1 shows the patient clinical characteristics at the beginning of the study. All the patients completed the 1-year follow-up.

**Table 1:** Distribution of the main clinical characteristics of the subjects at the beginning of the study

Clinical characteristic	Arthroplasty	NSAIDs	BIOF2	P
Men (%)	50.0%	62.5%	50.0%	0.84*
Age (years)	62.8 ± 8.6	68.0 ± 7.1	68.5 ± 8.9	0.34**
BMI	29.5 ± 2.9	29.8 ± 2.1	28.0 ± 3.3	0.41**
Smoking	37.5%	25.0%	25.0%	0.81*
Diabetes	37.5%	25.0%	12.5%	0.51*
High blood pressure	62.5%	37.5%	50%	0.60*
WOMAC	73.3 ± 15.2	61.6 ± 16.0	62.7 ± 21.6	0.366**
Rasmussen	15.0 ± 7.0	14.0 ± 2.2	12.7 ± 3.6	0.643**
RAPID3	7.0 ± 1.5	8.3 ± 0.6	7.4 ± 1.1	0.103**

Percentages or averages and standard deviation are shown. *BMI* body mass index.* Fisher’s exact test; **One-way ANOVA

**Fig. 1:**
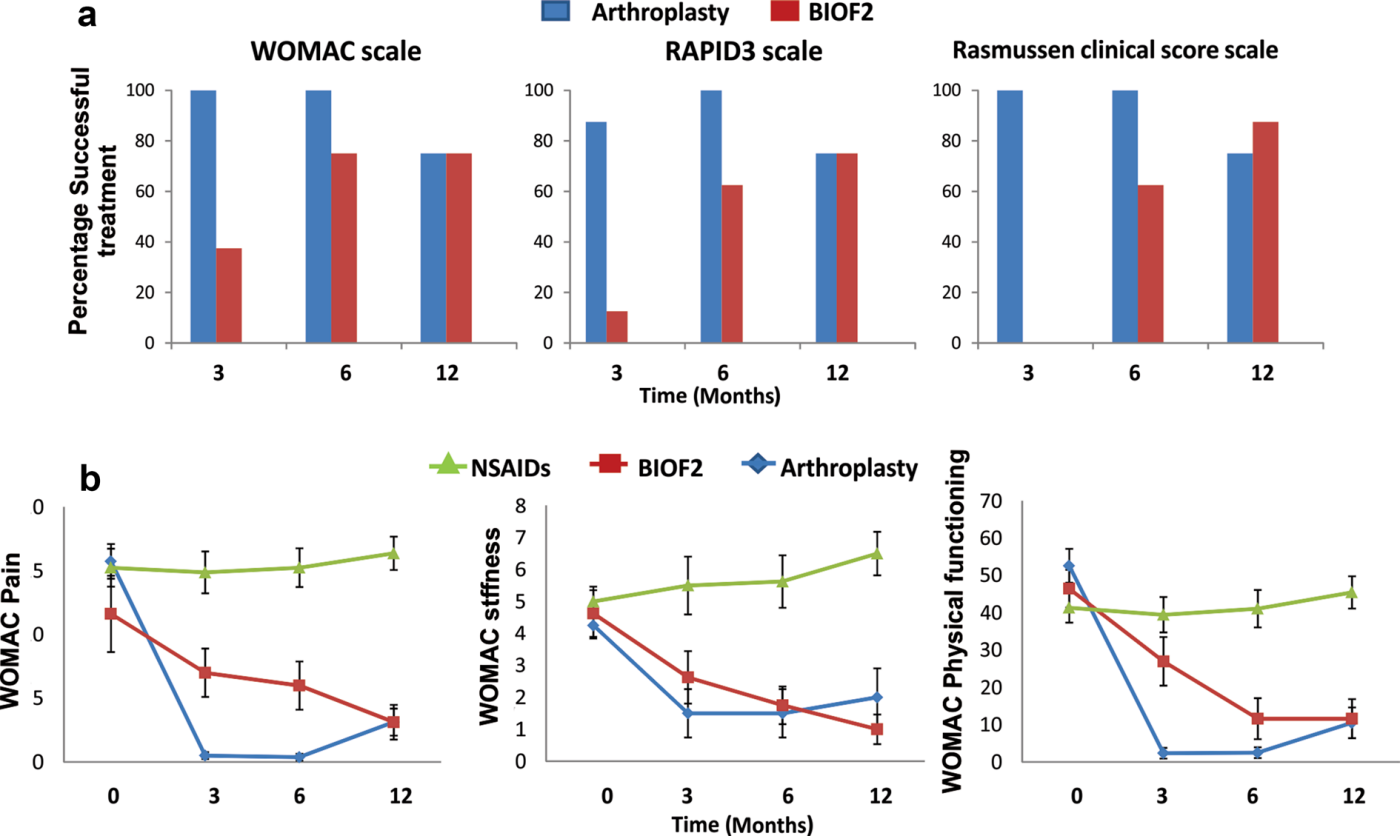
Clinical progression of patients over 12 months, according to the different scales for evaluating osteoarthritis. **a** Percentages of patients with successful treatment over time, according to the different evaluation scales are shown (scores: WOMAC ≤ 29 at 3 and 6 months and ≤ 16 points at 12 months, Rasmussen clinical score ≥ 24, RAPID3 ≤ 2). None of the patients in the NSAID group had treatment success. At month 6, 75% of the patients treated with BIOF2 had treatment success, according to the WOMAC scale. At month 12, the treatments with BIOF2 and arthroplasty showed no statistically significant differences, according to all the scales. **b** WOMAC subscales for pain, stiffness, and physical function. The group treated exclusively with NSAIDs had no changes over time. The treatments with BIOF2 and arthroplasty produced significant changes over time in all the subscales (one-way ANOVA test *P* < 0.05, for both groups). Mean and standard error were plotted

Figure 1a shows the success percentages with the different treatments. Arthroplasty reached high success rates at month 3 and both BIOF2 and arthroplasty had important success percentages starting at month 6 (success in ≥ 75% of the patients at month 6, according to the WOMAC score). At month 12, there were no statistically significant differences in the evaluated scores between the BIOF2 group and the arthroplasty group. Figure 1b shows patient progression according to the WOMAC subscales for pain, stiffness, and physical function. No changes in any of the subscales were produced over time in the NSAID group. In contrast, the BIOF2 group and arthroplasty group had significant improvement in the three subscales over time. Additional file 2 shows the values of the WOMAC, Rasmussen, and RAPID3 scores of the three groups throughout the 1 year of follow-up.

At the beginning of the study, 100% of the patients in the three groups required NSAIDs on a daily basis. They took from 2 to 3 different drugs to control their pain. Sixty-seven percent took paracetamol, 62% took diclofenac, 46% took ketorolac, 17% took naproxen, 17% took celecoxib, 8% took ibuprofen, and 12% took tramadol. At month 12 of the follow-up, 75% of the patients with BIOF2 and 75% of the patients with arthroplasty no longer required NSAID use. That reduction in NSAID consumption was statistically significant for both the BIOF2 group and the arthroplasty group, compared with the group that exclusively took NSAIDs (RR = 0.33, 95% CI 0.12–0.87, *P* = 0.02. This result was the same for BIOF2 vs NSAIDs and arthroplasty vs NSAIDs). At the end of the study, only two patients (25%) from the arthroplasty group and two patients (25%) from the BIOF2 group required habitual use of NSAIDs, whereas 100% the patients in the NSAID group required them daily.

**Fig. 2:**
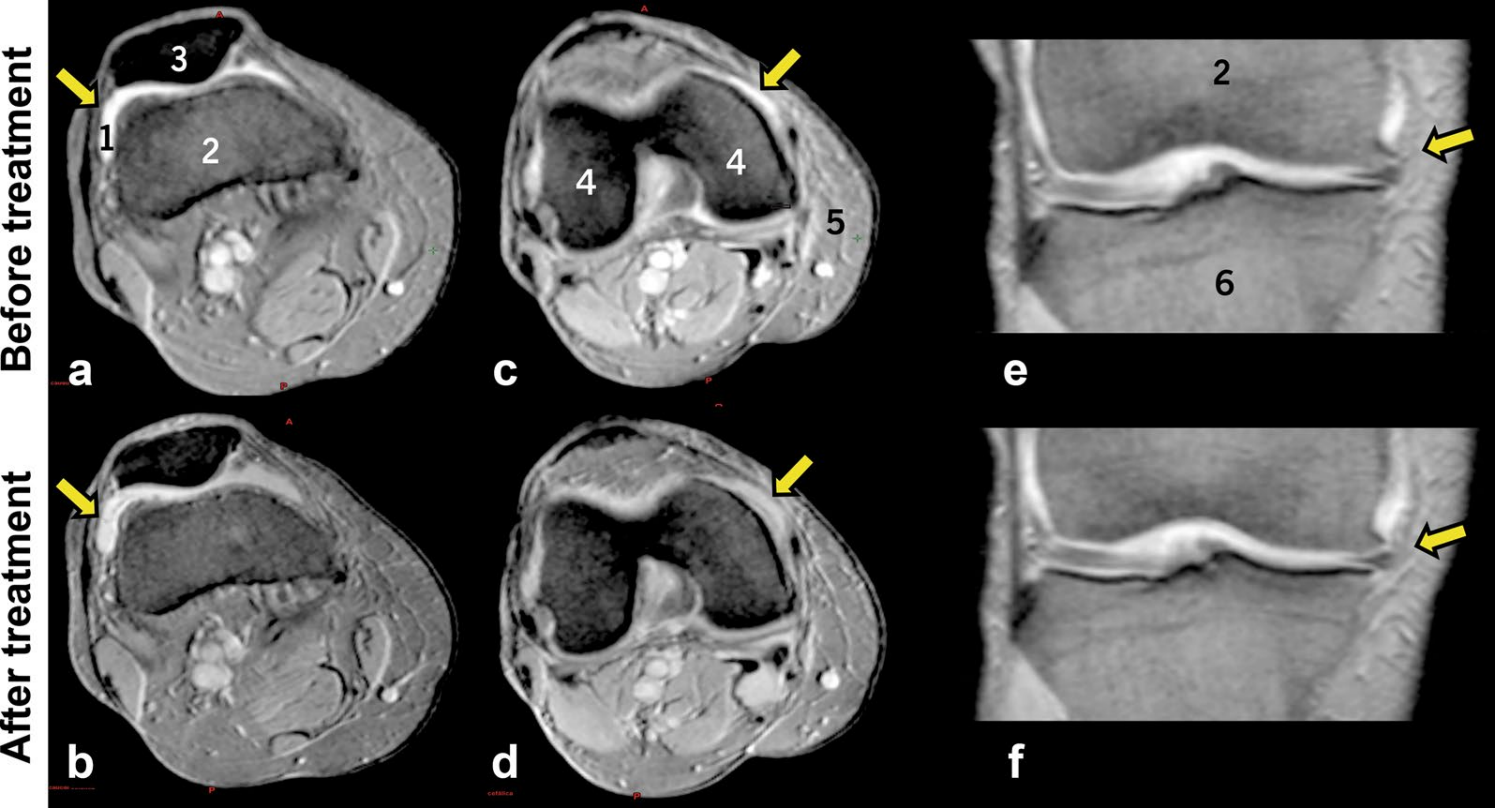
Nuclear magnetic resonance images of the knee joint before and after treatment with BIOF2. Axial views at the height of the patella (**a**, **b**) and the femoral condyles (**c**, **d**) and coronal views (**e**, **f**), before and after 12 months of treatment, respectively. The arrows indicate the zones in which treatment generated a beneficial change, with respect to cartilage thickness or continuity. 1: cartilage, 2: femur, 3: patella 4: femoral condyle, 5: muscle, 6: tibia

**Fig. 3:**
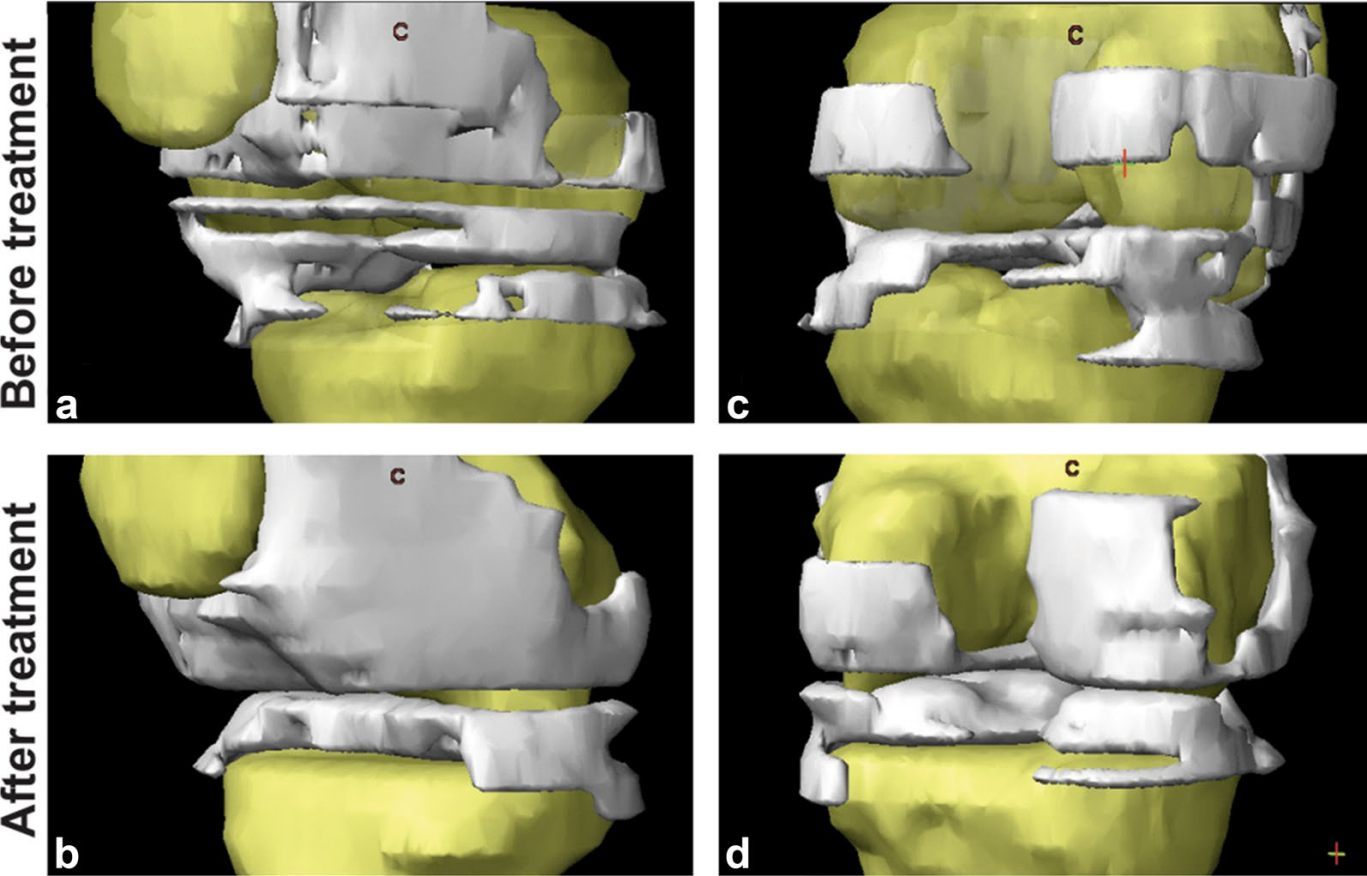
Three-dimensional reconstruction of the knee joint before and after 12 months of treatment with BIOF2. It shows the bone region (yellow) and cartilaginous region (white) of the joint in an anterolateral view (**a**, **b**) and a posterior view (**c**, **d**). The image corresponds to the patient that had the greatest increase in cartilage (43%). Femur and tibia cartilage fusion observed in some regions of the **a** or **b** images, corresponds to a defect in the three-dimensional reconstruction, in which the virtual space between the two structures, at that point in particular, was not able to be distinguished

Articular cartilage volume was a success parameter evaluated in the 8 patients of the BIOF2 group. Total articular cartilage was significantly increased by 22% at 1 year of treatment (26.16 ± 10.0 vs 31.96 ± 10.0 cm^2^, *P* < 0.001), with a range of 10 to 43%. The 6 patients with therapeutic success had at least a 20% increase in cartilage. The two patients in the BIOF2 group with failed treatment (according to the WOMAC score) were the patients that had the least increase of cartilage (18 and 10%). Even though those two patients did not reach scores considered therapeutic success, they reduced their WOMAC scores by 48 and 18%, respectively, at month 12. Figure 2 shows nuclear magnetic resonance images of the knee joint of the patient with the greatest cartilage growth, before and after treatment with BIOF2. Figure 3 shows the three-dimensional reconstruction of the knee of said patient. Articular cartilage volume was not correlated with the WOMAC score prior to treatment (*r* = − 0.37, *P* = 0.40) or at month 12 (*r* = − 0.46, *P* = 0.28). However, the percentage of increased cartilage had a reverse correlation with the WOMAC score at 12 months of treatment (*r* = − 0.75; *P* = 0.03). There was no correlation between the increase in cartilage and the RAPID3 score or the Rasmussen clinical score.

With respect to adverse effects, the patients presented with local pain in the joint after BIOF2 application of an intensity of 8.2 ± 0.4 (visual analogue scale of 0 to 10) and lasting 53 ± 45 s. In some cases, the pain radiated to the pelvis, but it ceded spontaneously. Toxicity tests were performed in the BIOF2 group at 72 h and at months 1, 3, 6, and 12. No serum alterations were found in the liver enzymes (ALT, AST, LDH, ALP, bilirubin, and albumin), glucose, creatinine, uric acid, urea, or electrolytes (Na, K, and Cl). The complete blood count showed leukocytes (due to neutrophils) above the normal limits in two patients at 72 h that returned to baseline levels at month 1 of treatment and was not associated with abnormal signs and symptoms. Serum inflammation markers decreased at 3 days of treatment, compared with their baseline levels and the erythrocyte sedimentation rate (286 ± 38 vs 249 ± 20, *P* = 0.001), fibrinogen (25.3 ± 5.0 vs 19.12 ± 6.0, *P* = 0.04), and C-reactive protein (1.6 ± 0.7 vs 1.1 ± 0.9, *P* = 0.01) returned to baseline values at month 1 of treatment. Interestingly, total cholesterol levels were significantly reduced, with respect to the baseline values, at 1 year of treatment (215 ± 40 vs 188 ± 8, *P* = 0.02), as were the triglyceride levels (240 ± 83 vs 151 ± 41, *P* = 0.01). The patients that underwent arthroplasty did not present with adverse effects, other than those normally expected after surgery. Six patients in the NSAID group (75%) presented with abdominal pain/discomfort at some point during follow-up, which was the reason the family physicians prescribed H2-blockers or proton pump inhibitors to all the patients of that group at the end of the follow-up period, to prevent severe acute NSAID-related gastroduodenal damage.

## Discussion

The intra-articular application of a new bioactive formulation, called BIOF2, demonstrated therapeutic efficacy that was clearly superior to conservative treatment with NSAIDs for the treatment of severe osteoarthritis of the knee. In addition, there was no statistically significant difference in its success rate, with respect to that of arthroplasty. Treatment success was accompanied by articular cartilage regeneration, with an increase of at least 20% in relation with pre-treatment cartilage volume. Therapeutic efficiency was tested through three scoring instruments that evaluated pain, functionality, and quality of life of the patients with osteoarthritis. Treatment with BIOF2 began to produce significant improvement at month 3.

It has previously been demonstrated in three different animal models that hyaline cartilage regeneration may be induced in vivo via the intra-articular application of BIOF2. A trial in an animal model showed that the thickness of the cartilage and the number of chondrocytes began to increase slightly on post-treatment day 14, and very significantly on day 28. Articular cartilage regeneration, stimulated by BIOF2, has now been demonstrated in humans. The cartilage of the knee increased 22% on average at month 12 of treatment, accompanied by significant clinical improvement starting at month 3. That is an important increase in cartilage. A previous study showed that when patients had severe osteoarthritis of the knee (identified through elevated WOMAC scores: 46 ± 6.2), they also presented with a rapid loss of articular cartilage mass (6.4 ± 0.7% at 12 months) [26]. Such cartilage loss is significantly higher than that experienced by patients with mild osteoarthritis [26]. Therefore, patients with severe osteoarthritis of the knee were catalogued as fast progressors in that study [26]. Our group treated with BIOF2 had WOMAC scores of 62.7 ± 21 at the beginning of the study, and so could be considered fast progressors [26]. Those patients did not lose cartilage, but rather had an increase of cartilage at month 12, showing that BIOF2 not only stopped the natural progression of the osteoarthritis, with respect to cartilage loss, but also reversed it to varying degrees.

Total cartilage volume did not correlate with the WOMAC score at the beginning or at the end of the study, but the cartilage increment percentage correlated with the WOMAC score at the end of the study. Thus, it can be supposed that the changes in articular cartilage volume were those related to the symptomatology of the patients and not to the net cartilage volume that the patient had at the end of treatment. The elevated correlation index between the post-treatment WOMAC score and the cartilage increment percentage could be clinically useful. In patients treated with cartilage regenerating substances, it could be assumed that a lower post-treatment WOMAC score would signify a greater increase in cartilage, eliminating the need for complex studies, such as nuclear magnetic resonance. Nevertheless, further studies on that topic are required.

BIOF2 is composed of a corticosteroid, a type of insulin, and organic acids. Corticosteroids are bioactive substances, but when acting alone, may facilitate tissue atrophy and joint destruction. However, when acting in synergy with the other factors in BIOF2, they can produce chondrogenesis. It has been proposed that BIOF2 modifies the intra-articular microenvironment to stimulate articular regeneration, by generating molecular and morphologic alterations in synovial fluid cells and chondrocytes. In human synovial cells, BIOF2 increases the expression of SOX9, a transcription factor that is essential for chondrocyte differentiation and cartilage formation [27, 28]. It also causes reduced expressions of the macrophage-stimulating protein receptor (MST1R) and mimecan (OGN). OGN has been reported to be elevated in osteoarthritis synovial fluid samples and may induce the mineralization and calcification of cartilage [29]. MST1R has been previously associated with osteoclastogenesis, osteolysis, and inflammation [30, 31]. The above data demonstrate a mechanism of action of BIOF2 that is consistent with the clinical results encountered.

It is clear that treatment of severe osteoarthritis of the knee with BIOF2 has advantages, compared with conservative treatment with NSAIDs, and could be an effective alternative to total joint arthroplasty. It is also a more economic and less complex procedure than arthroplasty, especially for patients of advanced age and/or those with comorbidities. In addition, BIOF2 significantly reduced NSAID use. Prolonged NSAID use can cause adverse effects, especially kidney damage [32]. Thus, treatment with BIOF2 could also help patients reduce the risks involved in the long-term use of those medications. Treatment with BIOF2 can be applied in an outpatient setting at a consultation office, taking the customary precautions for any intra-articular injection. The only adverse effect detected was pain upon application, and although intense, it spontaneously remitted within seconds or a little over a minute. Arthroplasty efficacy in our study was 75%. The panorama for improving quality of life is complex in patients with therapeutic failure after arthroplasty. In contrast, in patients with therapeutic failure after BIOF2 application, they could still opt to undergo the customary therapeutic alternatives, from viscosupplementation to arthroplasty.

Other clinical trials have evaluated articular cartilage regeneration through cellular therapy in the repair of defects in knee cartilage in young persons [33], in moderate osteoarthritis [34], in severe osteoarthritis [35], and in avascular bone necrosis [36] [37], mainly with positive results. Other procedures include implants utilizing novel biomaterials [38] and the use of genetically engineered chondrocytes [39], with varying degrees of effectiveness. However, unlike our study, none of those trials compared the efficacy of the procedures with total joint arthroplasty. Procedures with stem cells involve complex strategies that are costly and difficult to implement in medical centers. In addition, problems still exist that must be resolved in the future, including limited cell availability, the numerous surgical procedures involved, or in vitro chondrocyte dedifferentiation or cell propagation [11]. Therefore, we consider that treatment with the new bioactive cell-free formulation (BIOF2) is a promising and easily implemented option for the treatment of osteoarthritis or pathologies with articular cartilage loss due to other causes.

It is striking that in the patients treated with BIOF2, total cholesterol and triglyceride levels were significantly reduced at 1 year. It has been proposed that osteoarthritis of the knee affects mobility, mood, and sleep patterns, and reduces physical fitness, with an increase in cardiometabolic comorbidity [3, 5–7, 40]. Therefore, it is likely that patients treated with BIOF2, upon recovering their quality of life, have greater physical activity, thus reducing their cardiovascular risks, reflected in reduced serum lipids. However, a limitation of our study was the fact that serum markers or MRI evaluations were not carried out in the arthroplasty group or the NSAID group, and so we were not able to compare results with those of the BIOF2 group. The low number of patients and the 1-year follow-up period were other limitations of the present study and are aspects that must be considered in future analyses to broaden the knowledge about this new treatment.

## Conclusions

The intra-articular application of a new bioactive cell-free formulation, called BIOF2, was shown to be well tolerated, with a success rate that showed no statistically significant difference from that of arthroplasty for the treatment of severe osteoarthritis of the knee. Success is most likely related to articular cartilage regeneration. BIOF2 has great potential for use in osteoarthrosis as an easily implemented therapeutic alternative.

## Additional files

**Additional file 1.** CONSORT 2010 flow diagram.

**Additional file 2.** Comparison of the patient WOMAC score, Rasmussen clinical score, and RAPID3 score between groups before the intervention and at the successive months (mean ± standard deviation).

## Abbreviations

BIOF2: bioactive formulation for articular cartilage regeneration
NSAIDs: nonsteroidal anti-inflammatory drugs
RPCEC: Cuban Public Registry of Clinical Trials Database
MSCs: mesenchymal cells
WOMAC: Western Ontario and McMaster Universities Osteoarthritis Index
RAPID3: Routine Assessment of Patient Index Data 3
SOX9: SRY-Box 9 gene
MST1R: macrophage-stimulating protein receptor gene
OGN: mimecan gen
