# Supplementary material for: A phase I–II controlled randomized trial using a promising novel cell-free formulation for articular cartilage regeneration as treatment of severe osteoarthritis of the knee
Source: Eur J Med Res. 2018 Oct 24;23:52. doi: 10.1186/s40001-018-0349-2 (PMC6199741; doi:10.1186/s40001-018-0349-2)
Supplement: Supplementary file 1 — Additional file 1. CONSORT 2010 flow diagram. [file 40001_2018_349_MOESM1_ESM.doc]

**CONSORT 2010 Flow Diagram**

**Allocation**

**Analysis**

**Follow-Up**

**Enrollment**

Assessed for eligibility (n= 58 )

Excluded (n= 34 )

♦  Not meeting inclusion criteria (n=20)

♦  Declined to participate (n=14)

♦  Other reasons (n= 0)

Analysed (n=8)
♦ Excluded from analysis (give reasons) (n=0)

Lost to follow-up (give reasons) (n=0)

Discontinued intervention (give reasons) (n=0)

Allocated to BIOF2 intervention (n=8)

♦ Received allocated intervention (n=8)

♦ Did not receive allocated intervention (n= 0)

Lost to follow-up (give reasons) (n=0)

Discontinued intervention (give reasons) (n=0)

Allocated to Arthroplasty intervention (n=8)

♦ Received allocated intervention (n=8)

♦ Did not receive allocated intervention (n=0)

Analysed (n=8)
♦ Excluded from analysis (give reasons) (n=0)

Randomized (n=24)

Lost to follow-up (give reasons) (n=0)

Discontinued intervention (give reasons) (n=0)

Allocated to conservative intervention with NAIDs (n=8)

♦ Received allocated intervention (n=8)

♦ Did not receive allocated intervention (n=0)

Analysed (n=8)
♦ Excluded from analysis (give reasons) (n=0)
