# Supplementary material for: A phase I–II controlled randomized trial using a promising novel cell-free formulation for articular cartilage regeneration as treatment of severe osteoarthritis of the knee
Source: Eur J Med Res. 2018 Oct 24;23:52. doi: 10.1186/s40001-018-0349-2 (PMC6199741; doi:10.1186/s40001-018-0349-2)
Supplement: Supplementary file 2 — Additional file 2. Comparison of the patient WOMAC score, Rasmussen clinical score, and RAPID3 score between groups before the intervention and at the successive months (mean ± standard deviation). [file 40001_2018_349_MOESM2_ESM.docx]

Comparison of the patient WOMAC score, Rasmussen clinical score, and RAPID3 score between groups before the intervention and at the successive months (mean ± standard deviation)

| **Group** | **Time** | | | |
| --- | --- | --- | --- | --- |
|  | Baseline | Month 3 | Month 6 | Month 12 |
|  | **WOMAC** | | | |
| NSAIDs | 61.6±16.0 | 59.8±20.1 | 62.0±20.6 | 68.3±17.0 |
| Arthroplasty | 73.3±15.2 | 4.3±5.4 | 4.3±5.4 | 15.6±15.8 |
| BIOF2 | 62.7±21.6 | 36.6±25.2 | 19.3±18.9 | 15.7±17.6 |
| **P ANOVA** | **0.366** | **< 0.001** | **< 0.001** | **< 0.001** |
|  | **Rasmussen** | | | |
| NSAIDs | 14.0±2.2 | 13.8±1.9 | 14.2±2.1 | 13.8±1.8 |
| Arthroplasty | 15.0±7.0 | 27.7±1.8 | 27.8±1.9 | 26.3±3.9 |
| BIOF2 | 12.7±3.6 | 16.1±5.4 | 23.7±3.4 | 25.7±2.3 |
| **P ANOVA** | **0.643** | **< 0.001** | **< 0.001** | **< 0.001** |
|  | **RAPID3** | | | |
| NSAIDs | 8.3±0.6 | 8.3±0.6 | 8.4±0.6 | 8.6±0.4 |
| Arthroplasty | 7.0±1.5 | 0.7±1.0 | 0.4±0.5 | 0.6±1.1 |
| BIOF2 | 7.4±1.1 | 3.9±1.7 | 2.6±2.2 | 1.5±1.9 |
| **P ANOVA** | **0.103** | **< 0.001** | **< 0.001** | **< 0.001** |
|  |  | **Post hoc Analysis*** | |  |
| **WOMAC** |  |  |  |  |
| BIOF2 vs NSAIDs | 1.000 | 0.068 | < 0.001 | < 0.001 |
| BIOF2 vs Plasty | 0.742 | 0.008 | 0.247 | 1.000 |
| **Rasmussen** |  |  |  |  |
| BIOF2 vs NSAIDs | 1.000 | 0.648 | < 0.001 | < 0.001 |
| BIOF2 vs Plasty | 1.000 | < 0.001 | 0.014 | 1.000 |
| **RAPID3** |  |  |  |  |
| BIOF2 vs NSAIDs | 0.399 | < 0.001 | < 0.001 | < 0.001 |
| BIOF2 vs Plasty | 1.000 | < 0.001 | 0.017 | 0.591 |

* Bonferroni's post hoc test
